# The effect of a kindergarten-based, family-involved intervention on objectively measured physical activity in Belgian preschool boys and girls of high and low SES: the ToyBox-study

**DOI:** 10.1186/1479-5868-11-38

**Published:** 2014-03-14

**Authors:** Marieke De Craemer, Ellen De Decker, Maïté Verloigne, Ilse De Bourdeaudhuij, Yannis Manios, Greet Cardon

**Affiliations:** 1 Department of Movement and Sport Sciences, Ghent University, Watersportlaan 2, 9000 Ghent, Belgium; 2 Department of Nutrition and Dietetics, Harokopio University, E. Venizelou 70, 17671 Athens, Greece

**Keywords:** Physical activity, Kindergarten, Accelerometer, Intervention effect, Children

## Abstract

**Background:**

The ToyBox-study developed an evidence- and theory-based intervention to improve preschoolers’ energy balance-related behaviours – including physical activity (PA) – by targeting the kindergarten environment and involving their parents/caregivers. The present study aimed to examine the effect of the ToyBox-intervention on increasing Belgian preschoolers’ objectively measured PA levels.

**Methods:**

A sample of 472 preschoolers (4.43 ± 0.55 years; 55.1% boys) from 27 kindergartens (15 intervention, 12 control kindergartens) in Flanders, Belgium were included in the data analyses. Preschoolers wore an ActiGraph accelerometer for six consecutive days and were included in the data analyses if they had a minimum of two weekdays and one weekend day, both at baseline and follow-up (one year later). Preschoolers’ PA outcomes were estimated for an average day, weekday, weekend day, during school hours, and during after school hours. To assess intervention effects, multilevel repeated measures analyses were conducted for the total sample, and for sub-groups (according to sex, kindergarten levels of socio-economic status (SES) and risk groups (low levels of PA at baseline)) of preschoolers.

**Results:**

Small intervention effects were found in the total sample. Most intervention effects were found in boys and in preschoolers from high SES kindergartens. Boys from the intervention group had an increase in vigorous PA (ß = 1.47, p = 0.03) and moderate-to-vigorous PA (ß = 1.27, p = 0.03) from baseline to follow-up, whereas PA levels in boys from the control group stagnated or decreased. In preschoolers from high SES kindergartens, the largest effects were found for PA outcomes during school hours and during after school hours.

**Conclusion:**

The results from the Belgian sample demonstrate that effects of the PA-component of the ToyBox-intervention on objectively measured PA were found in preschool boys and in preschoolers from high SES kindergartens, which means that the ToyBox-intervention was mainly effective in those sub-groups. Future interventions should search for alternative strategies to increase preschoolers’ PA levels in preschool girls and preschoolers from low SES kindergartens, as these are the most important at-risk groups regarding PA.

## Introduction

Preschool children (between four and six years old) should engage in sufficient levels of physical activity (PA), because even at this young age, PA is associated with a number of positive mental and physical health outcomes [1,2]. Furthermore, childhood PA tracks from year to year [3-5]. PA also plays an important role in the prevention of overweight and obesity in children [1,6]. Nevertheless, most preschoolers accumulate low PA levels throughout the day [7], and few preschool children comply with the current PA guidelines for preschoolers of 180 minutes of total PA per day [7-9]. In addition, according to the review of Hinkley et al. (2008), already at preschool age differences in physical activity levels between boys and girls exist, with preschool boys being more physically active compared to preschool girls [10], and preschool boys engaging in activities with higher intensities compared to preschool girls [7,11]. Furthermore, while one study found more higher intensity PA in preschoolers’ from families with higher socio-economic status (SES) [12], recent systematic reviews found no association between preschoolers’ SES and PA levels [10,11].

Several studies have focused on increasing preschoolers’ PA levels. As preschool children spend a considerable amount of time at some form of out-of-home care, these settings provide an opportunity to increase preschoolers’ PA, resulting in preschool-based interventions [13]. Some studies investigated the effect of targeting preschoolers’ PA levels during recess, but mixed results were found [14-16]. Activity-friendly equipment during recess increased three- to five-year-old US preschoolers’ PA [16], whereas providing four- to five-year-old Belgian preschoolers with play equipment and playground markings did not increase PA levels [15]. In addition, lowering the playground density resulted in only small improvements in four- to six-year-old Belgian preschoolers’ activity levels [14]. Other preschool-based interventions targeted the preschool curriculum to increase preschoolers’ PA levels [17]. For example, teacher-led structured PA sessions, integrated in the preschool curriculum, are promising to increase PA in four- to six-year-old Belgian preschoolers [13]. A study in three- to five-year-old US preschoolers consisted of a curriculum of 18 weeks with 15- to 20-minute-lessons – comprising of multiple activities focusing on stability (trunk strength), locomotor skills (running, hopping, skipping), or manipulation skills (ball skills) – four days a week (72 lessons in total). Although positive changes in gross motor skills were found, no intervention effects were found for PA [17]. To conclude, only a few preschool-based interventions in preschool children have been successful in increasing preschoolers’ PA levels.

Involving parents to increase preschoolers’ PA levels may be promising [18-23], because children also spend – next to the out-of-home care – a considerable amount of time at the home environment, and significant correlations exist between child’s PA level and parental support [24]. The involvement of parents in interventions with preschoolers is still understudied. However, one study of O’Dwyer et al. (2012) demonstrated a significant increase in three- to five-year-old English preschoolers’ PA on week (4.5%) and weekend days (13.1%), after their family received a ten-week active play program [25].

Also combined interventions exist – a preschool-based and a parental involvement component – targeting an increase in preschoolers’ PA levels, but inconsistent results were found [26,27]. In the study of Reilly et al. [26] in four-year-old Irish preschoolers, PA programs (i.e., three 30-minute sessions per week for 24 weeks) and home-based health education (i.e., families receiving materials and health education leaflets) did not increase PA. In another study, parents were motivated to develop and implement their own project ideas to promote four- to six-year-old German preschoolers’ PA, apart from a state-sponsored program consisting of gym classes twice a week for six months [27]. Results showed that preschool children in the intervention group had an increase in total PA, compared to the control group in which no increase was found [27].

The ToyBox-study aimed at preventing overweight and obesity in four- to six-year-old preschoolers, by developing a theory- and evidence-based multidisciplinary kindergarten intervention with family involvement and testing it in six European countries (Belgium, Bulgaria, Germany, Greece, Poland, and Spain) [28]. The evidence-based kindergarten intervention with family involvement was framed in a social ecological perspective because of the significant influence of the family environment and individual factors. The intervention was developed with the use of the PRECEDE-PROCEED model [29] and the Intervention Mapping protocol [30], and targets four behaviours: (1) PA, (2) sedentary behaviour, (3) water consumption, and (4) snacking.

The present study aimed to examine the effect of the ToyBox-intervention on four- to six-year-old preschoolers’ objective PA levels in Belgium, as only Belgian preschoolers wore accelerometers to objectively measure PA. Consequently, differences in light PA (LPA), moderate PA (MPA), vigorous PA (VPA), total PA, and moderate-to-vigorous PA (MVPA) were investigated from baseline to follow-up. In addition, we examined whether the intervention had a different impact on PA in boys versus girls, in low versus high SES kindergartens, or in low (high levels of PA at baseline) versus high risk groups (low levels of PA at baseline).

## Methods

### Study protocol

The kindergarten-based ToyBox-intervention with family involvement – targeting four- to six-year-old preschoolers – had a randomized cluster design and consisted of a pre-test post-test design with intervention and control schools across six European countries. Preschool children and their families were recruited at kindergartens, daycare centers or preschool settings. In order to avoid confusion for the reader, these settings will be referred to as “kindergartens” in this paper. Only in Belgium, preschoolers’ PA levels were measured by the use of accelerometers. Since these motion sensors assess different intensities of objectively-based PA, the effect of the ToyBox-intervention on light, moderate, as well as vigorous PA of Belgian preschool children could be evaluated.

**Figure 1:**
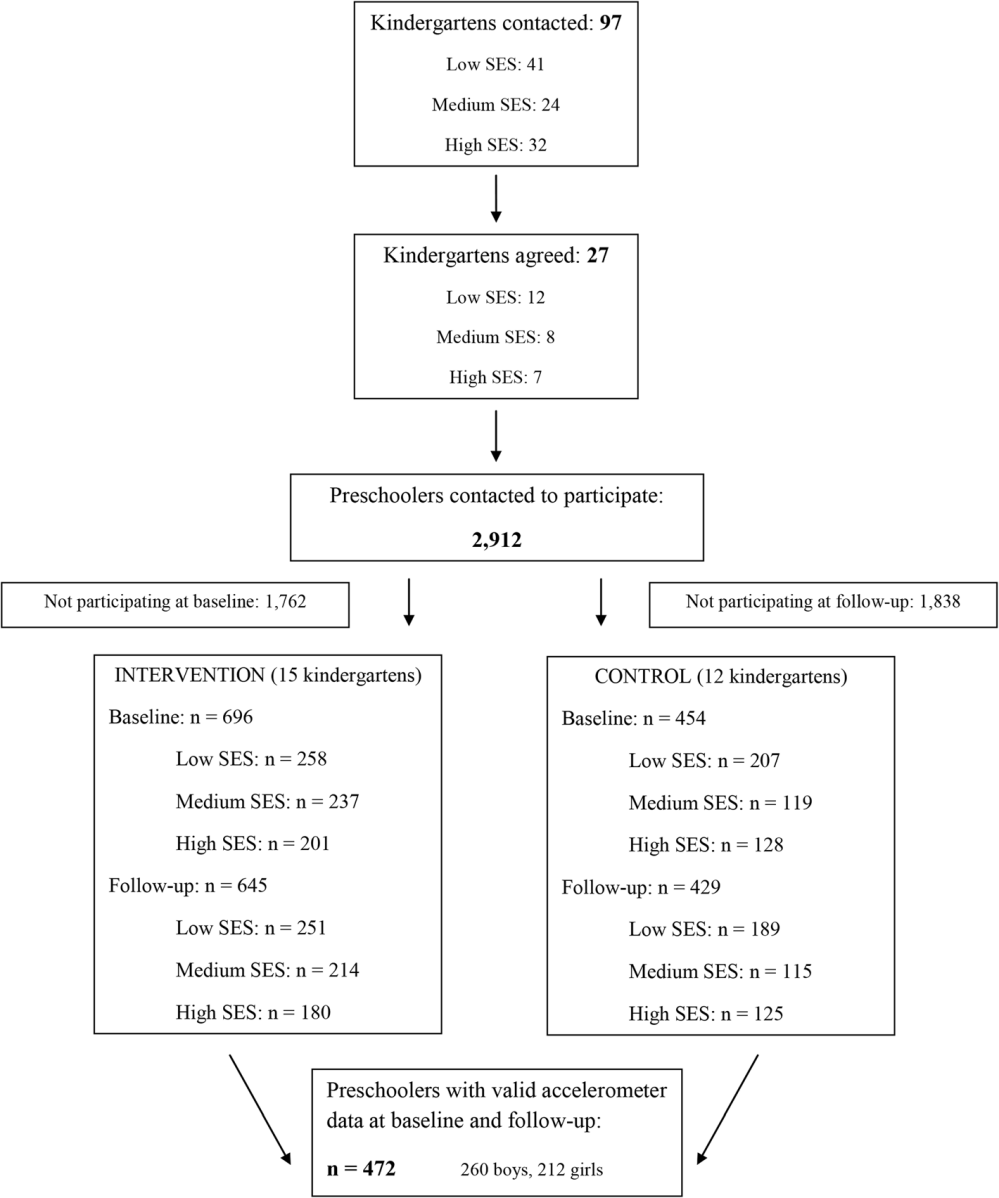
**Flow chart of included kindergartens and preschoolers in the ToyBox-study.** SES = Socio-economic status.

Within two provinces (West- and East-Flanders) in Flanders, the northern part of Belgium, kindergartens were recruited from different socio-demographic backgrounds. Lists of all municipalities that exist within the selected provinces were collected and information on the SES variables was provided (mean years of education for the population of 25–55 years or annual income). Tertiles were created, based on the selected SES variables, and five municipalities were randomly selected from those tertiles (i.e., five municipalities for low SES, five for medium SES, and five for high SES). Then, 97 kindergartens within these randomly chosen municipalities were randomly selected (with the exclusion of the lowest 20% of the smallest kindergartens), and a personal visit was performed to inform the kindergarten staff about the ToyBox-study. Twenty-seven kindergartens (27.8%) agreed to participate in the study, and all preschoolers of the first and second kindergarten class (n = 2,912) received an information letter to take home in which the purpose of the study was explained to the parents/caregivers, and the child was invited to wear an accelerometer for six consecutive days (Figure 1).

After the recruitment of the kindergartens, kindergartens’ municipalities were randomly assigned to the intervention or the control condition (2:1) to avoid contamination between kindergartens in the same municipality. Kindergartens allocated to the intervention condition received the intervention material which could be used during the school year 2012–2013 (from October 2012 until March 2013). Kindergartens allocated to the control condition were informed that they would receive the intervention material one year later (which they could use to their own needs), and that they could continue with the normal kindergarten curriculum.

Before the start of the intervention, pre-test measurements were performed on weekdays from March until June 2012. On those days, researchers visited intervention and control kindergartens and fitted those preschool children with an accelerometer for whom written informed consent from their parents/caregivers had been obtained. One year later, from March until June 2013, post-test measurements were performed and again, preschoolers with written informed consent from both intervention and control kindergartens received an accelerometer to objectively measure their PA levels. The Belgian part of the ToyBox-study was approved by the Ethical Committee of the Ghent University Hospital (EC/2010/037).

### ToyBox-intervention: PA-component

The kindergarten-based ToyBox-intervention with family involvement was planned and developed following the PRECEDE-PROCEED model [29] and the six different steps of the Intervention Mapping protocol (i.e., Needs assessment, Formulation of change objectives, Selection of theory-based methods and practical strategies, Development of the intervention and materials, Development of an adoption and implementation plan, Evaluation planning) [30], and included the four different behaviours which are aimed at in the ToyBox-study [28]. These energy balance-related behaviours were handled in four different components, including the PA-component.

The ToyBox-intervention consisted of 24 intervention weeks, and started in October 2012 until March 2013. Before the start of the ToyBox-intervention, environmental changes for PA occurred in the classroom, which were retained throughout the whole school year. The PA-part of the intervention was implemented in weeks 5 until 8, and had a two-week repetition period in weeks 19 and 20. During the other weeks, the other three behaviours were targeted and implemented. Furthermore, some PA-components were also implemented throughout the whole school year. The ToyBox-intervention was implemented by the kindergarten teachers, who had two teachers’ training sessions with the researchers to explain the goals and the material of the ToyBox-study, and to answer to kindergarten teachers’ questions, prior to the intervention. During the first training session (i.e., before the start of the intervention), teachers were provided with the “ToyBox”, a box containing a teachers’ guide, classroom activity guides, newsletters, tip-cards, posters and a kangaroo hand puppet. The teachers’ guide provided some background information, for example on the definition of PA and the importance of increasing preschoolers’ PA levels to establish healthy PA behaviour. In the classroom activity guide for PA, three different themes were included, namely (1) setting environmental changes in the classroom (i.e., how to rearrange the classroom; this was retained throughout the whole school year), (2) the child performing the actual behaviour (i.e., being physically active during structured PA sessions; these sessions were implemented for 20 weeks), and (3) classroom activities (i.e., kangaroo stories, and PA excursions; these activities were implemented for six weeks in total). Before the start of the repetition period, teachers received a third teachers’ training, during which the goals of the ToyBox-intervention were repeated and a short repetition was provided concerning the behaviours and the materials. Teachers were asked to allocate a minimum of one hour per week to use the ToyBox-materials and to implement the ToyBox-intervention in the classroom. To involve the parents/caregivers, preschoolers received two newsletters, two tip-cards and one poster (with key messages on PA that could be colored at kindergarten or at home) to take home for their parents/caregivers. The newsletters and tip-cards contained tips and strategies to increase preschoolers’ PA levels.

To get an insight in the role of the main implementers and their fidelity in implementing the ToyBox-intervention (i.e., teachers and parents/caregivers), process evaluation tools were developed. Teachers received monthly logbooks, containing questions on changes made to the kindergarten environment, preschoolers’ performing PA, execution of classroom activities, whether they handed out the intervention materials and what their feedback was on the intervention materials. At the end of the ToyBox-intervention, preschoolers’ parents/caregivers received a questionnaire containing questions on whether they received and read the newsletters, tip-cards and poster, and how they perceived these materials (e.g., reliable, understandable, useful).

### Instrumentation

To objectively quantify Belgian preschoolers’ PA levels, three models of ActiGraph accelerometers (Pensacola, FL) were used, namely the GT1M (3.8 cm × 3.7 cm × 1.8 cm; 27 g), the GT3X (3.8 cm × 3.7 cm × 1.8 cm; 27 g), and the GT3X + (4.6 cm × 3.3 cm × 1.5 cm; 19 g). The use of three different accelerometer models was inevitable as all available accelerometers had to be used in order to measure a large sample in a limited amount of time. Accelerometers were worn on the right hip, secured by an elastic waist band. Only the vertical axis output was used in the present study. There is a strong agreement between the GT1M, GT3X and GT3X + accelerometers, which makes it acceptable to use these activity monitors together in one study [31]. Furthermore, the GT1M accelerometer has been validated to measure PA in preschool children [32]. Accelerometers were initialized to measure activity counts in 15-second epochs – because of preschoolers’ intermittent pattern of movement [33] – using ActiLife version 5.5.5-software.

### Procedure

Preschoolers’ parents/caregivers were instructed to let their child wear the accelerometer during all waking hours for six consecutive days, including two weekend days, and only to remove the device during water-based activities and during sleeping. To ensure that the accelerometer was worn correctly, parents/caregivers were given an informational letter with instructions on how to handle the device.

After data collection, accelerometers were downloaded and raw data files were then reduced using the Meterplus version 4.3 software (Santech Inc., San Diego, US). Both the first and sixth day were omitted, because these days were incomplete. Periods of ten minutes or more of consecutive zeros were deleted, as these periods were regarded as non-wearing time. To be included in the analyses, preschoolers were required to have at least six hours of accelerometer recordings on two weekdays and one weekend day [34]. For ease of interpretation, the 15-second data were divided by four to express the outcome in minutes. Minutes of LPA, MPA, and VPA were afterwards categorized using the cut-points by Evenson et al. [35], which are recommended to use in this age group [36]. The procedure of data collection, data deposition and data reporting was standardized and harmonized within the ToyBox-intervention.

### Statistical analyses

For PA, all outcomes (i.e., LPA, MPA, VPA, total PA, and MVPA) were separately calculated for an average day, weekday, weekend day, during school hours (between 8 AM and 4 PM), and after school hours (between 4 PM and 8 PM). To take into account that some preschoolers had more weekend days than others, outcome variables on an average day were calculated using the following formula: ((MEAN (outcome on weekday 1, outcome on weekday 2)*5) + (MEAN(outcome on weekend day 1, outcome on weekend day 2))/7. All outcomes were expressed in percentages of the total wearing time by dividing all outcome variables by the total wearing time and multiplying by 100. Prior to all analyses, all outcome measures were first checked for normal distribution (skewness < 0.70). Several outcome measures were positively skewed and were therefore logarithmically transformed (log_10_). Transformed variables were used in the analyses, but for ease of interpretation, non-transformed data were reported in the text and tables. Descriptive statistics were computed to describe the characteristics (age, sex, SES) of the sample, and were reported as percentages or means and standard deviations.

Multilevel repeated measures analyses were performed using MLwiN 2.28 (Centre for Multilevel Modelling, University of Bristol, UK) to assess the effectiveness of the intervention on all outcome variables [37]. Multilevel modeling (four levels: time; preschool child; kindergarten class; kindergarten) was used to take clustering of two measurements (baseline and follow-up) of preschool children in kindergarten classes in kindergartens into account. The likelihood ratio test was used to justify that the data fits the model. All analyses were adjusted for age and sex. Two ß-values will be reported in the results: (1) the ß-value for ‘time’ is the estimate for the time effect, and can be interpreted as the magnitude of change in the outcome variable going from baseline to follow-up, irrespective of the condition to which preschoolers belong, and (2) the ß-value for ‘time*condition’ is the estimate for the intervention effect for all outcome variables, which describes the difference between the mean change in the intervention group and the mean change in the control group. To investigate the differences between boys and girls, different levels of SES from the kindergarten, and differences between low risk (i.e., preschoolers with the highest two tertiles of total PA at baseline) and high risk (i.e., preschoolers with the lowest tertile of total PA at baseline) groups, three-way interaction effects (‘time*condition*sex’, ‘time*condition*SES’, and ‘time*condition*risk group’) were calculated for all outcome variables. When these three-way interaction effects were significant, stratifications in sub-groups were reported. To consider the effect size of significant intervention effects, we reported Cohen’s d statistic (small = 0.20, moderate = 0.50, large = 0.80). Cohen’s d statistic was calculated using the means and standard deviations from the intervention and control group (d = (M_1_ – M_2_) / √[($s_{1}^{2}$ + $s_{2}^{2}$)/2]) [38]. Values were only reported in the text, not in the tables. To check for differences between the intervention and control group at baseline, multilevel regression analyses were conducted (three-level: preschool child; kindergarten class; kindergarten). To compare the preschool children who had valid data with the preschool children who did not have valid data, attrition analyses were conducted as a three-level logistic regression analysis (preschool child; kindergarten class; kindergarten). For all analyses, statistical significance level was set at p < 0.05, and p-values between 0.05 and 0.10 were considered borderline significant.

## Results

In total, 472 preschoolers (55.1% boys, mean age 4.43 ± 0.55 years) provided valid accelerometer data at baseline and follow-up. The flow of participants through the study is illustrated in Figure 1. The mean accelerometer wearing time was 11.8 ± 1.1 hours and 12.1 ± 3.3 hours per day for baseline and follow-up respectively. At baseline, preschoolers spent 54.3% (± 6.4) of the day in total PA. At follow-up, they spent 54.5% (± 6.2) of the day in total PA. For all dependent variables, no significant differences were found between the intervention and control group at baseline. Attrition analyses showed that preschool girls were more likely to have incomplete data than preschool boys (OR = 1.16; 95% CI = −0.11 – 0.42), and older preschool children were more likely to have incomplete data than younger preschool children (OR = 1.48; 95% CI = 0.13 – 0.69).

Results obtained from the multilevel repeated measures analyses for the PA outcomes in the total sample are shown in Table 1. For VPA and MVPA during after school hours, a significant intervention effect was found with preschoolers from the intervention group having an increase in VPA and MVPA from baseline to follow-up, and preschoolers from the control group having a decrease in VPA (ß = 1.31, p = 0.04, d = 0.14) and MVPA (ß = 1.18, p = 0.03, d = 0.16). Two borderline significant intervention effects were found for VPA on a weekday and MVPA on an average day, with preschoolers from the intervention group having a steeper increase in VPA on a weekday (ß = 1.14, p = 0.09, d = 0.01) and MVPA on an average day (ß = 1.07, p = 0.09, d = 0.12) from baseline to follow-up, compared to the control group.

**Table 1:** Time and interaction effects for PA outcomes in the total sample (adjusted for age and sex)

**n = 472 (I = 301, C = 171)**		**PRE (%)**	**POST (%)**	**Time**	Time * condition
				**ß**	**ß**
**LPA**					
Day	I	47.7	46.8	−0.80^*^	−0.06
	C	46.7	45.9		
Weekday	I	48.1	47.1	−1.18^**^	0.11
	C	47.4	46.2		
Weekend day	I	46.5	46.3	−0.11	−0.09
	C	45.2	45.1		
School hours^$^	I	49.0	39.6	−1.26^***^	1.02
	C	47.8	37.8		
After school hours	I	38.8	34.1	−5.86^***^	1.18
	C	38.3	32.4		
**MPA**					
Day^$^	I	5.6	6.3	1.05^*^	1.06
	C	5.6	5.9		
Weekday^$^	I	5.5	6.1	1.05	1.05
	C	5.5	5.8		
Weekend day^$^	I	5.6	6.1	1.05	1.02
	C	5.4	5.7		
School hours	I	5.9	6.0	−0.33	0.45
	C	6.0	5.6		
After school hours^$^	I	4.3	4.2	1.15^**^	1.11
	C	4.3	3.7		
**VPA**					
Day^$^	I	1.7	2.1	1.16^**^	1.11
	C	1.8	2.0		
Weekday^$^	I	1.5	1.9	1.16	1.14^*^
	C	1.5	1.8		
Weekend day^$^	I	1.8	2.3	1.17	1.08
	C	1.9	2.2		
School hours^$^	I	1.2	1.7	1.43^***^	1.02
	C	1.2	1.8		
After school hours^$^	I	1.2	1.5	−1.02	1.31^**^
	C	1.3	1.3		
**Total PA**					
Day	I	55.5	56.0	−0.05	0.48
	C	54.6	54.6		
Weekday	I	55.7	55.9	−0.48	0.65
	C	55.2	54.7		
Weekend day	I	55.1	55.9	0.93	−0.09
	C	53.6	54.6		
School hours^$^	I	56.4	48.5	−1.19^***^	1.03
	C	55.2	46.2		
After school hours	I	45.6	41.5	−6.06^***^	1.93
	C	45.2	39.2		
**MVPA**					
Day^$^	I	7.4	8.6	1.08^**^	1.07^*^
	C	7.5	8.1		
Weekday^$^	I	7.0	8.1	1.08	1.07
	C	7.2	7.8		
Weekend day^$^	I	7.7	8.7	1.09	1.04
	C	7.6	8.2		
School hours^$^	I	6.8	7.6	1.06	1.05
	C	6.8	7.3		
After school hours^$^	I	5.7	6.1	−1.10	1.18^**^
	C	5.9	5.4		

*p < 0.10, **p < 0.05, ***p < 0.001.

^$^Variable was log-transformed.

LPA = Light Physical Activity; MPA = Moderate Physical Activity; VPA = Vigorous Physical Activity; Total PA = Total Physical Activity; MVPA = Moderate- to Vigorous Physical Activity; I = Intervention group; C = Control group.

A significant three-way interaction effect was found for sex for LPA during school hours (p = 0.04), and also a borderline significant interaction effect was found for sex for total PA during school hours (p = 0.07). This means that changes in PA between the intervention and control group from baseline to follow-up were different for boys and girls. Results obtained from the multilevel repeated measures analyses for the PA outcomes stratified by sex are illustrated in Table 2. In boys, significant intervention effects were found for VPA and MVPA during after school hours. For VPA during after school hours, boys from the intervention group had a 0.5% increase from baseline to follow-up, whereas VPA in boys from the control group stagnated (ß = 1.47, p = 0.03, d = 0.26). Furthermore, boys from the intervention group had a 0.9% increase in MVPA during after school hours from baseline to follow-up, whereas boys from the control group had a 0.5% decrease (ß = 1.27, p = 0.03, d = 0.19). Finally, a borderline significant intervention effect was found in boys for VPA on a weekday, with boys from the intervention group having a steeper increase compared to boys from the control group (ß = 1.20, p = 0.09, d = 0.10). In girls, only borderline significant intervention effects were found for LPA and total PA during school hours. Girls from the control group had a steeper decrease in LPA (ß = 1.14, p = 0.06, d = 0.30) and total PA (ß = 1.11, p = 0.06, d = 0.31) during school hours from baseline to follow-up, compared to girls from the intervention group who had a smaller decrease.

**Table 2:** Time and interaction effects for LPA, MPA, VPA, total PA, and MVPA in boys and girls (adjusted for age)

		**Boys n = 260 (I = 168, C = 92)**				**Girls n = 212 (I = 133, C = 79)**			
		**PRE (%)**	**POST (%)**	**Time**	Time * condition	**PRE (%)**	**POST (%)**	**Time**	Time * Condition
				**ß**	**ß**			**ß**	**ß**
		**LPA**							
Day	I	47.8	46.8	−0.46	−0.64	45.7	45.1	−1.22^*^	0.64
	C	46.2	45.7			45.5	44.3		
Weekday	I	48.2	46.9	−0.78	−0.56	46.1	54.4	−1.64^**^	0.91
	C	46.9	46.1			46.1	44.5		
Weekend day	I	46.4	46.3	0.42	−0.46	45.3	44.9	−0.73	0.34
	C	44.8	44.4			44.3	43.5		
School hours^$^	I	49.4	38.6	−1.21^***^	−1.06	46.7	39.9	−1.33^***^	1.14^*^
	C	47.5	39.5			46.1	34.6		
After school hours	I	38.9	34.0	−5.20^***^	0.32	37.7	33.3	−6.63^***^	2.21
	C	37.9	32.7			37.7	31.1		
		**MPA**							
Day^§^	I	5.7	6.4	1.04	1.09	5.5	6.1	0.42^*^	0.17
	C	5.6	5.8			5.5	5.9		
Weekday^§^	I	5.5	6.2	1.05	1.08	5.5	6.0	0.32	0.19
	C	5.5	5.8			5.4	5.7		
Weekend day^$^	I	5.7	6.2	1.04	1.06	5.3	5.6	1.08	−1.03
	C	5.4	5.6			5.2	5.6		
School hours^§^	I	5.4	5.4	−1.06	1.06	5.4	5.6	−0.38	0.50
	C	5.5	5.2			5.4	5.0		
After school hours^$^	I	4.3	4.4	−1.14	1.18	4.3	3.9	−1.15^*^	1.02
	C	4.2	3.7			4.4	3.8		
		**VPA**							
Day^$^	I	1.7	2.1	1.14^*^	1.10	1.7	2.3	1.19^**^	1.13
	C	1.8	2.0			1.9	2.3		
Weekday^$^	I	1.5	2.0	1.12	1.20*	1.6	2.0	1.22^**^	1.07
	C	1.5	1.7			1.7	2.1		
Weekend day^$^	I	1.9	2.2	1.19	1.00	1.9	2.6	1.14	1.18
	C	1.9	2.3			2.0	2.3		
School hours^$^	I	1.2	1.8	1.38^**^	1.08	1.4	1.9	1.48^***^	−1.05
	C	1.2	1.7			1.4	2.1		
After school hours^$^	I	1.2	1.7	−1.02	1.47^**^	1.3	1.5	−1.01	1.13
	C	1.2	1.2			1.6	1.6		
		**Total PA**							
Day	I	55.7	55.9	0.17	−0.01	53.1	53.8	−0.34	1.01
	C	54.1	54.3			53.1	52.8		
Weekday	I	55.8	55.8	−0.16	0.18	55.5	55.7	−0.86	1.15
	C	54.8	54.6			55.5	54.7		
Weekend day	I	55.1	56.0	1.44	−0.49	53.3	53.9	0.36	0.32
	C	53.2	54.7			52.3	52.6		
School hours^$^	I	56.9	47.5	−1.15^***^	−1.04	53.8	48.3	−1.24^***^	1.11^*^
	C	55.2	47.9			53.2	42.9		
After school hours	I	45.7	41.5	−5.12^**^	0.97	44.5	40.4	−7.15^***^	3.03
	C	44.7	39.6			44.9	37.8		
		**MVPA**							
Day^§^	I	7.5	8.7	1.06	1.09	7.4	8.7	0.90^*^	0.36
	C	7.4	7.9			7.6	8.5		
Weekday^§^	I	7.1	8.3	1.07	1.10	7.3	8.4	0.79^**^	0.25
	C	7.2	7.7			7.5	8.2		
Weekend day^$^	I	8.0	8.7	1.09	1.02	7.3	8.4	1.09	1.05
	C	7.5	8.2			7.4	8.0		
School hours^$^	I	6.8	7.7	1.05	1.07	6.5	7.2	1.07	1.04
	C	6.9	7.2			6.5	7.0		
After school hours^$^	I	5.6	6.5	−1.10	1.27^**^	5.9	5.8	−1.09	1.07
	C	5.7	5.2			6.2	5.7		

*p < 0.10, **p < 0.05, ***p < 0.001.

^$^Variable was log-transformed for both boys and girls.

^§^Variable was log-transformed for boys.

LPA = Light Physical Activity; MPA = Moderate Physical Activity; VPA = Vigorous Physical Activity; Total PA = Total Physical Activity; MVPA = Moderate- to Vigorous Physical Activity; I = Intervention group; C = Control group.

Significant three-way interaction effects were found for kindergarten SES for several PA outcomes (p < 0.05), meaning that changes in PA between the intervention and control group from baseline to follow-up were different for kindergarten SES levels. Results obtained from the multilevel repeated measures analyses for the PA outcomes stratified by SES are depicted in Table 3. For preschoolers from low SES kindergartens, no positive intervention effects – with the exception of VPA during after school hours – were found between the intervention group and the control group from baseline to follow-up. For preschoolers from medium SES kindergartens, a significant intervention effect was found for LPA on a weekday, with preschoolers from the intervention group having a smaller decrease in LPA from baseline to follow-up compared to the control group (ß = 2.35, p = 0.03, d = 0.12). For preschoolers from high SES kindergartens, significant intervention effects were found for LPA, MPA, and total PA during school and after school hours, for MPA and MVPA on an average day and on a weekday and for VPA on a weekday. Preschoolers from the intervention group had a smaller decrease in LPA (ß = 1.36, p < 0.001, d = 0.81) and total PA (ß = 1.27, p < 0.001, d = 0.65) during school hours from baseline to follow-up compared to the control group. The same results were found for LPA (ß = 7.97, p < 0.001, d = 0.74) and total PA (ß = 9.52, p < 0.001, d = 0.75) during after school hours. Furthermore, preschoolers from the intervention group had a 1.0% and 1.2% increase in MPA on an average day (ß = 1.21, p = 0.004, d = 0.61) and on a weekday (ß = 1.26, p = 0.002, d = 0.58) whereas the control group stagnated and had a 0.2% decrease respectively. For MVPA on an average day, preschoolers from the intervention group had a 1.2% increase from baseline to follow-up, whereas preschoolers from the control group only had a 0.1.0% increase in MVPA (ß = 1.21, p = 0.01, d = 0.56). For MVPA on a weekday, preschoolers from the intervention group had an increase in MVPA whereas the control group decreased (ß = 1.29, p = 0.004, d = 0.52). Also, for VPA on a weekday, preschoolers from the intervention group increased their VPA whereas preschoolers from the control group stagnated from baseline to follow-up (ß . 1.42, p = 0.02, d = 0.32).

**Table 3:** Time and interaction effects for LPA, MPA, VPA, total PA, and MVPA in preschoolers from low SES, medium SES and high SES kindergartens (adjusted for sex and age)

		**Low SES n = 169 (I = 102, C = 67)**				**Medium SES n = 149 (I = 104 , C = 45)**				**High SES n = 154 (I = 95, C = 59)**			
		**PRE (%)**	**POST (%)**	**Time**	Time * condition	**PRE (%)**	**POST (%)**	**Time**	Time * condition	**PRE (%)**	**POST (%)**	**Time**	Time * condition
				**ß**	**ß**			**ß**	**ß**			**ß**	**ß**
**LPA**													
Day	I	47.0	46.4	−0.04	−0.52	48.9	47.0	−3.00^***^	1.08	46.7	46.4	0.08	−0.38
	C	45.5	45.4			49.2	46.2			45.7	45.8		
Weekday	I	48.0	47.0	−0.13	−0.95	48.8	47.8	−3.33^***^	2.35^**^	46.9	45.9	−1.09	0.04
	C	46.5	46.4			49.6	46.3			46.4	45.3		
Weekend day	I	44.8	45.3	0.63	−0.10	48.1	45.6	−3.04^**^	0.49	46.6	47.7	1.53	−0.44
	C	43.1	43.7			47.6	44.6			45.5	47.0		
School hours^1^	I	49.6	39.6	−1.07	−1.17^**^	49.8	38.6	−1.25^**^	1.04	48.1	41.3	−1.58^***^	1.36^***^
	C	47.4	44.5			50.9	40.9			45.8	29.0		
After school hours	I	38.1	35.1	−3.26^**^	0.26	38.7	31.3	−1.98	−5.40^*^	39.3	35.5	−11.80^***^	7.97^***^
	C	38.2	34.9			39.0	37.1			37.2	25.4		
**MPA**													
Day^2^	I	5.8	6.3	0.23	0.32	5.8	6.3	1.16^**^	−1.07	5.7	6.7	−1.01	1.21^**^
	C	5.7	5.9			5.5	6.4			5.9	5.9		
Weekday^2^	I	5.8	6.1	0.28	−0.01	5.6	6.1	1.20^**^	−1.10	5.4	6.6	−1.04	1.26^**^
	C	5.7	6.0			5.5	6.5			5.8	5.6		
Weekend day^3^	I	5.2	5.6	1.09	−1.01	6.0	6.6	0.12	0.41	6.2	6.7	1.06	1.01
	C	5.0	5.4			5.9	6.0			6.0	6.4		
School hours^4^	I	5.9	5.8	−0.21	0.08	5.6	5.3	1.09	−1.13	5.9	6.5	−0.95^**^	1.64^***^
	C	5.9	5.7			5.6	6.1			5.9	5.0		
After school hours^1^	I	4.5	4.0	−1.14	1.03	4.3	4.1	1.11	−1.15	4.3	4.5	−1.37^**^	1.41^**^
	C	4.1	3.6			4.2	4.7			4.5	3.3		
**VPA**													
Day^1^	I	1.6	2.1	1.14	1.14	1.6	2.0	1.33^**^	−1.05	1.9	2.4	1.03	1.26^*^
	C	1.8	2.0			1.5	2.1			1.9	1.9		
Weekday^1^	I	1.4	1.8	1.09	1.12	1.4	1.9	1.54^***^	−1.14	1.6	2.2	−1.01	1.42^**^
	C	1.6	1.7			1.3	2.0			1.7	1.7		
Weekend day^1^	I	1.7	2.4	1.26^*^	1.09	1.6	1.9	−1.00	1.21	2.3	2.7	1.17	1.01
	C	1.9	2.4			1.8	1.8			1.9	2.2		
School hours^1^	I	1.2	1.5	1.26^**^	1.05	1.1	1.8	1.55^***^	1.05	1.3	1.9	1.58^***^	−1.09
	C	1.2	1.5			1.2	1.8			1.3	2.1		
After school hours^1^	I	1.2	1.4	−1.29^*^	1.50^**^	1.1	1.9	1.38	1.25	1.3	1.4	1.05	1.04
	C	1.3	1.0			1.1	1.5			1.5	1.5		
**Total PA**													
Day	I	54.5	55.2	0.65	−0.004	57.1	56.1	−1.62	0.60	54.8	56.1	0.21	1.12
	C	53.2	53.8			57.0	55.4			54.0	54.2		
Weekday	I	55.6	55.3	0.52	−0.78	56.7	56.6	−1.58	1.52	54.6	55.4	−1.28	2.08
	C	54.0	54.6			57.0	55.4			54.6	53.4		
Weekend day	I	52.9	54.7	2.02	−0.30	56.2	54.6	−2.81	1.19	56.3	58.3	2.84^*^	−0.76
	C	51.1	53.1			56.0	53.2			54.4	57.3		
School hours^1^	I	56.6	47.9	−1.05	−1.12^**^	57.3	47.9	−1.18^**^	−1.01	55.9	50.6	−1.40^***^	1.27^***^
	C	54.6	51.8			58.6	49.7			53.6	38.2		
After school hours	I	45.0	42.2	−3.66^*^	0.91	45.7	50.7	−0.85	5.82^*^	46.2	43.0	−12.66^***^	9.52^***^
	C	44.9	41.2			45.6	44.7			44.6	32.0		
**MVPA**													
Day^2^	I	7.5	8.7	0.67^*^	0.53	7.4	8.4	1.20^**^	−1.06	7.7	9.3	1.00	1.21^**^
	C	7.8	8.4			7.1	8.6			7.9	8.0		
Weekday^1^	I	7.0	7.5	1.05	1.02	7.1	8.1	1.27^***^	−1.11	7.1	8.9	−1.02	1.29^**^
	C	7.0	7.4			6.8	8.7			7.7	7.5		
Weekend day^1^	I	7.1	8.2	1.13	1.02	7.3	8.2	1.00	1.12	8.9	9.8	1.12	−1.01
	C	7.1	8.0			7.5	7.6			8.1	9.0		
School hours^1^	I	6.7	7.0	1.03	1.01	6.8	7.6	1.18^**^	−1.05	6.8	8.2	1.03	1.16
	C	6.6	6.8			6.9	8.2			7.0	7.2		
After school hours^1^	I	5.7	5.8	−1.20^**^	1.22^*^	5.6	6.6	1.15	1.02	5.8	6.1	−1.15	1.22
	C	5.7	5.7			5.6	6.4			6.1	5.4		

*p < 0.10, **p < 0.05, ***p < 0.001.

^1^Variable was log-transformed for all three SES-levels.

^2^Variable was log-transformed for medium SES and high SES.

^3^Variable was log-transformed for low SES and high SES.

^4^Variable was log-transformed for medium SES.

LPA = Light Physical Activity; MPA = Moderate Physical Activity; VPA = Vigorous Physical Activity; Total PA = Total Physical Activity; MVPA = Moderate- to Vigorous Physical Activity; I = Intervention group; C = Control group; SES = Socio-Economic Status.

No significant three-way interaction effects were found with baseline PA level, meaning that changes in PA between the intervention and control group from baseline to follow-up were not different for high risk groups (low levels of PA at baseline) and low risk groups (high levels of PA at baseline). Therefore, no risk group-stratifications were performed.

## Discussion

The aim of the present study was to examine the effect of the ToyBox-intervention on Belgian four- to six-year-old preschoolers’ objective PA levels. Differences in PA outcomes were investigated from baseline to follow-up. Kindergarten-based and family-involved components were used in the intervention to achieve the goal of increasing preschoolers’ PA levels. At the kindergarten level, teachers had to implement the PA intervention component and received a manual in which environmental changes in the classroom, PA sessions, and classroom activities were described. To involve parents/caregivers at the home environment, educational materials (newsletters, tip-cards, poster) were provided to the parents/caregivers to introduce them with strategies and tips and tricks to increase their child’s PA levels. It was expected that the intervention would increase preschoolers’ PA of different intensities, whereas PA levels from preschoolers from the control group would stagnate.

In the total sample, intervention effects were found for VPA and MVPA during after school hours. There was a 0.3 and 0.4% increase in the intervention group for VPA and MVPA during after school hours compared to a stagnation in VPA and a 0.5% decrease in MVPA in the control group. Taking an accelerometer wearing time of four hours during after school hours into account, this 0.3% and 0.4% increase in VPA and MVPA corresponds to an additional 0.7 and 0.9 minutes of VPA and MVPA during after school hours. These small increases in VPA and MVPA during after school hours were also reflected in the small effect sizes. Additionally, borderline significant effects were found for VPA on a weekday and MVPA on an average day, with the intervention group having a 0.4% increase in VPA and a 1.2% increase in MVPA. Assuming an average accelerometer wearing time of ten hours per day, this 0.4% and 1.2% increase in VPA and MVPA respectively, corresponds to an additional 2.4 and 7.2 minutes of VPA and MVPA. Although these intervention effects for the higher intensities of PA were statistically significant in the total sample, the biological relevance should be interpreted with caution since it is unclear whether this small increase in VPA and MVPA will cause a health effect in preschool children.

Further, changes in PA between the intervention group and control group from baseline to follow-up were different for boys and girls. During after school hours, boys from the intervention group had a 1.0% increase in time spent in VPA and MVPA whereas time spent in VPA and MVPA in boys from the control group stagnated and declined, respectively. The increase in higher intensities of PA in preschool boys was very small and was again reflected in the small effect sizes. In preschool girls, only borderline significant effects were found for total PA and LPA during school hours, with girls from the intervention group having a smaller decrease in time spent in total PA and LPA during school hours. This means that stronger intervention effects were found in boys compared to girls, which might indicate that more effort should be taken to involve preschool girls in PA interventions. A possible strategy might be to make changes in the current ToyBox-material to make it more attractive to preschool girls. At the moment, the classroom activity guide for PA consisted for a significant part of structured PA sessions which mostly consisted of higher intensity activities. Since preschool boys engage in activities with higher intensities compared to preschool girls [7,11] and preschool boys are found to be more physically active in general [10], the ToyBox-material might have addressed preschool boys more compared to preschool girls.

Different effects were found for preschoolers from high versus low SES kindergartens, which means kindergartens located in high versus low SES neighbourhoods. In preschoolers from low SES kindergartens, negative intervention effects were found for time spent in total PA and LPA during school hours, with preschoolers from the intervention group having a steeper decrease from baseline to follow-up compared to the control group who had a smaller decrease in total PA and LPA. In contrast with the negative intervention effects in low SES kindergartens, we did find positive intervention effects for all PA outcomes in high SES kindergartens. Since kindergarten SES was based on SES of the municipality, it might be plausible to say that low or high SES kindergartens were located in low or high SES neighbourhoods. In high SES neighbourhoods, children have more opportunities to be physically active because of a higher access to a private garden at home and the availability of safe playgrounds in the neighbourhood [39]. Children from low SES neighbourhoods have less access to a private garden at home, a park or suitable nearby nature [39]. Furthermore, playgrounds in low SES neighbourhoods are more hazardous compared to playgrounds from high SES neighbourhoods [39]. Therefore, it might have been easier for preschoolers’ parents/caregivers from high SES neighbourhoods to put the information from the newsletters and the tips and strategies from the tip-cards into practice. Furthermore, parents/caregivers from high SES neighbourhoods might perceive the traffic as safer [40], and together with the information from the newsletters and tip-cards this might have increased preschoolers’ active transportation to high SES kindergartens. Although the effects of low and high SES neighbourhoods on PA are understudied in this age group, this might be a possible explanation for the positive intervention effects on preschoolers’ PA from high SES kindergartens only.

In preschool girls and high SES kindergartens, most intervention effects were found for PA outcomes during school and after school hours. In girls and in high SES kindergartens, preschoolers from the intervention group had a smaller decrease in total PA and LPA during school hours compared to the control group. This decrease in total PA and LPA might be explained by the fact that preschool children have to learn to sit still in preparation of primary school [41], which might have caused a shift from total PA and LPA to sedentary time. However, this decrease was smaller in the intervention group in girls and children from high SES kindergartens, which means that the intervention had a positive effect on the decrease in total PA and LPA in girls and preschoolers from high SES kindergartens, and the implementation of the intervention might have counteracted the steep decrease of time spent in total PA and LPA during school hours. Furthermore, half of the preschool children at follow-up were going to the third kindergarten class (birth year 2007), which means that – in Belgium – they get the opportunity to participate in after school activities (i.e., structured activities out of school like preschool gymnastics, swimming classes, football). Since one study found a decline in physical activity when preschool children get older [42] and PA rapidly declines during childhood and adolescence [43], the participation in after school activities in older preschool children might have slowed down the decrease in LPA and total PA during after school hours in the intervention group.

Although Belgian preschoolers’ physical activity levels were measured during the same period (March-June 2012) at baseline and at follow-up (one year later; March-June 2013), the weather might partly explain the decrease in physical activity levels of preschoolers from the control group. During Spring 2012, the mean outside temperature was 10.5°C, whereas this was only 7.7°C during Spring 2013. In addition, there were more hours of sunshine during Spring 2012 compared to Spring 2013 (469 hours vs. 386 hours), and less rainy days (43 days vs. 47 days) with less rain in total (200.4 mm vs. 222.5 mm) (http://www.meteo.be). Other studies have also reported higher physical activity levels in preschool children when the weather is warmer or drier compared to colder and wetter weather conditions [44,45].

A possible explanation for the fact that only small effects were found for the total sample of preschoolers after receiving the ToyBox-intervention might be that Belgian (Flemish) kindergartens already implement PA components into the curriculum. For example, preschoolers already receive structured PA sessions during the time they spend at kindergarten [46]. In Flemish kindergartens, these sessions are scheduled in the curriculum for two hours per week to realize one of the developmental goals of the kindergarten curriculum prescribed by the Flemish government, namely physical education of the preschool child [13,47]. In the ToyBox-intervention, preschool teachers had to implement the PA-module for at least one hour per week, which is shorter compared to the physical education sessions preschoolers already receive. This might mean that the intervention dose might have been too low to cause more effect in the total sample, as both intervention and control groups already received the two hours of physical education in the curriculum.

A detailed process-evaluation on the implementation of the PA-component of the ToyBox-intervention by both the teachers and the parents/caregivers might be needed to provide insights in and to draw meaningful conclusions on the outcome results. For example, an explanation for the limited intervention effects in the total sample could be the lack of kindergarten teachers’ motivation to spend time on increasing preschoolers’ PA levels due to time constraints and the full curriculum. Another explanation might be related to the implementation of the parental-involved component of the ToyBox-intervention. Preschoolers’ parents/caregivers received two newsletters, two tip-cards and a poster with tips and tricks to increase their child’s PA levels. Nevertheless, it might be possible that part of the preschoolers’ parents/caregivers did not read the newsletters and tip-cards, or that they did not carry out the tips and tricks at home. Since only materials were handed out to the parents/caregivers, there was only a passive parental-involved component in the ToyBox-intervention. In addition, actively involving parents/caregivers as intervention targets might be a promising factor in an intervention [18,19,22,23]. Thus, intensifying the parental-involved component of the ToyBox-intervention possibly might lead to better effects. Finally, the time spent on PA during the ToyBox-intervention might have been too short (six weeks in total, but with the environmental changes implemented throughout the whole school year and the structured PA sessions implemented for 20 weeks) and/or the intensity might have been too low (a minimum of one hour per week) to expect changes in preschoolers’ PA levels. The short time and the low intensity spent on PA had two reasons. First of all, the ToyBox-intervention targeted four different behaviours, of which PA was only one. Therefore, only a limited amount of time (six weeks for each behaviour) was available to focus on preschoolers’ PA. Secondly, to enhance future implementation of the ToyBox-intervention, teachers had to implement the intervention instead of researchers. Consequently, the time asked to allocate to each of the intervention components was kept to a minimum, so that the implementation was more attainable for the teachers.

Study limitations include the use of different accelerometer models, and the relatively large drop-out of children due to the lack of valid accelerometer data. Strengths of the present study include the objective assessment of PA in a large sample of four- to six-year-old Belgian preschoolers, with the disposal of different PA intensities, and the randomized controlled trial with the pre-test post-test design including an intervention and control group.

## Conclusion

The ToyBox-intervention caused small effects in the total sample, but the biological relevance should be interpreted cautiously. Further, positive effects were found in preschool boys and in preschoolers from high SES kindergartens, meaning that the ToyBox-intervention was mainly effective in these sub-groups. These results show that preschoolers’ sex and kindergartens’ SES are moderators of the intervention effects on preschoolers’ objectively measured PA. Future interventions should search for alternative strategies to target preschool girls and preschoolers from low SES kindergartens to increase PA levels in these sub-groups, as these are at-risk groups for PA. In addition, future research could focus on examining the environmental details of the neighbourhoods in which the kindergartens were located and how this is related to preschoolers’ PA.

## Abbreviations

PA: Physical activity; SES: Socio-economic status; LPA: Light physical activity; MPA: Moderate physical activity; VPA: Vigorous physical activity; MVPA: Moderate-to-vigorous physical activity.

## Competing interests

The authors have no competing interests to declare.

## Authors’ contributions

All authors read and approved the final manuscript, and participated in the ToyBox-project. MDC and EDD were responsible for the data collection. MDC wrote the manuscript.
